# Caregiving and COVID-19: Perspectives from a Care Coach

**DOI:** 10.1093/geroni/igab046.3365

**Published:** 2021-12-17

**Authors:** Kelly Marnfeldt, Lilly Estenson, Julia Rowan, Kathleen Wilber

**Affiliations:** 1 University of Southern California, Los Angeles, California, United States; 2 University of Southern California, University of Southern California, California, United States

## Abstract

Family caregivers of community-dwelling older adults have faced unprecedented caregiving challenges during the COVID-19 pandemic. Examining the accumulated impact on family caregivers can help health and aging service providers design resources and supports that are resilient to emergency situations, and reduce negative psychological and physical consequences and risk of abuse within caregiving dyads. Data was collected as part of a pilot intervention in which “Care Coaches” provided telephonic coaching sessions to family caregivers of older adults. We examined Care Coach observations documented after coaching sessions with 24 family caregivers between March 2020 and February 2021. Two coders employed thematic analysis to generate codes and themes. The sample was 70% female, 80% were the spouse or significant other of their care receiver, the mean age was 61, and 53% were Non-Hispanic White. Themes and sub-themes included: (1) increased caregiver burden and diminished care networks due to fear of exposure to or contraction of COVID-19, (2) barriers to accessing in-home personal assistance services and home-delivered meals despite intervention efforts, and (3) the exacerbation of caregiver social isolation due to COVID-19 lockdown policies. Findings highlight the ways in which COVID-19 has amplified caregiver burden through the breakdown of formal and informal support systems. Potential adaptations of community-based services for older adults and their caregivers include remote service liaisons and need assessment of caregiver dyads to assure access to home-based personal assistance services and nutrition support for those at greatest risk of negative consequences during emergency service lapses.

